# New Rehabilitation Concept for Maxillary Edentulism: A Clinical Retrospective Study of Implant Crown Retained Removable Partial Dentures

**DOI:** 10.3390/jcm10081773

**Published:** 2021-04-19

**Authors:** Soo-Yeon Yoo, Seong-Kyun Kim, Seong-Joo Heo, Jai-Young Koak, Hye-Rin Jeon

**Affiliations:** 1 Department of Prosthodontics and Dental Research Institute, Seoul National University Dental Hospital, School of Dentistry, Seoul National University, 101 Daehak-ro, Jongno-gu, Seoul 03080, Korea; sy0502@snu.ac.kr (S.-Y.Y.); 0504heo@snu.ac.kr (S.-J.H.); young21c@snu.ac.kr (J.-Y.K.); 2 Department of Mathematics, Ewha Womans University, Seoul 03760, Korea; katehyerinjeon@gmail.com

**Keywords:** implant-crown-retained removable partial denture (IC-RPD), implant overdenture (IOD), survival rate, marginal bone loss (MBL), patient reported oral measurements (PROMs), prosthetic complications

## Abstract

There have been no studies of implant-crown-retained removable partial dentures (IC-RPD) for the treatment of maxillary edentulism. The purpose of this study was to perform clinical and radiographic evaluations of implants in IC-RPD compared to implant overdentures (IOD) in maxillary edentulous patients. Twenty IC-RPDs with 74 splinted implant crowns and 18 IODs with 71 implants retained with magnet attachments were observed in 38 patients. We statistically analyzed survival rates and marginal bone loss (MBL) of implants based on multiple variables including first year pathologic condition, location of placed implant, age, and sex in both treatments. Patient reported oral measurements (PROMs) regarding functional/esthetic improvement after IC-RPD or IOD treatments and prosthetic complications were also statistically analyzed. After a median observation period of 47.1 months (up to 147 months), we observed 97.3% implant survival rates for IC-RPD and 70.4% for IOD (*p* < 0.001). Among variables, first year pathologic condition (*p* < 0.001) and sex (*p* = 0.027) influenced implant survival rates. The MBL of implants for IC-RPD and IOD groups at the final check-up were 1.12 ± 1.19 mm and 3.31 ± 1.71 mm, respectively (*p* < 0.001). In both groups, patients with peri-implantitis (*p* < 0.001) and patients older than 65 years (*p* = 0.029) showed significantly higher implant MBL regardless of treatment modality. Functional and esthetic satisfaction were significantly improved (*p* < 0.001) after both treatments. The IOD group showed more frequent prosthetic complications compared to the IC-RPD group. Within the limitations of a retrospective study, we concluded that RPD with few splinted implant crowns is a feasible alternative treatment modality for maxillary edentulous patients with anatomical limitations.

## 1. Introduction

The conventional treatment modality for edentulous maxilla is conventional dentures (CD), but for several decades, implant dentistry has been able to restore edentulous maxillae with full fixed prostheses. Implant prosthodontic treatment in the edentulous maxilla is challenging due to anatomical limitations such as reduced alveolar bone and pneumatized paranasal sinuses. Additionally, socioeconomic limitations of patients and difficulties of zygomatic implant surgeries or lateral sinus floor elevation often prevent extensive surgery in the resorptive maxilla. In such cases, clinicians may consider restoring the edentulous maxilla with implant overdentures (IOD) using a few implants without bone augmentation or additional surgeries. Goodacre et al. reported that the highest failure rate (21.3%) for any type of prosthesis is associated with maxillary IOD [1]. This low success rate is attributed primarily to bone quality in the edentulous maxilla, since a looser arrangement of trabecular bone with a thin, or even absent cortical plate, is generally less capable of stabilizing and supporting implants [2]. The divergent implant axis due to adverse ridge morphology in the maxilla might cause excessive prosthesis loading.

To overcome these problems, the placement of more implants and implant splinting are recommended [3]. However, fixed prosthesis rehabilitation with sufficient implant number may require excessive surgeries and cost in maxillary edentulous patients. Therefore, the application of few splinted bar implants in IOD may be a feasible alternative [4,5,6]. Maxillary bar-retained IOD have been studied extensively, and a high implant survival rate of 96.3–98.2% per year was reported in a previous study [7].

In previous reports, the survival of IOD implants was ascribed to bar splinting [4,8,9,10,11]. A prospective study by Naert et al. found that the cumulative survival rate (CSR) of implants was 88.6% for IOD with bar type attachments, but only 40% for IOD with either balls or magnets after a mean loading time of 6.4 years [8]. The favorable results for bar IOD in maxillary edentulous patients were attributed to the number of implants that were rigidly splinted. However, splinting implants with a bar also introduces problems including the need for sufficient inter-occlusal space, difficulty in maintenance, un-esthetic morphology, soft tissue hyperplasia, and increased cost [12,13,14,15,16,17]. For fabrication of bar attachment, excellent skill and high cost are needed. Therefore, for the limitations and complications of bar fabrication in IOD, some clinicians suggest that IOD could be retained with sufficient solitary implant attachments for greater retention and support compared to CD [18,19,20,21,22]. A study of Kenney et al. [23] evaluated the photo elastic stress patterns produced by IODs. They found that independent solitary attachments transferred less stress to implants than the splinted bar attachments.

In a previous study, four un-splinted implants showed high short-term implant survival [18]. Tarnow et al. reported five consecutive cases illustrating that un-splinted (solitary) maxillary implants can be used to retain maxillary IOD [19]. These results agree with those of a 10-year study demonstrating high maxillary implant survival rates for IOD installed by both splinted and un-splinted methods using only three maxillary implants [20]. A longitudinal prospective study [21] of 49 patients with IOD found no significant difference in implant survival rate between IOD groups restored with ball (solitary) or round-bar attachments (bar splinted). Consequently, positive results have been reported for IOD using un-splinted implants [22].

However, a systematic review suggested that IOD with non-splinted anchorage and ≤4 implants increased risk of implant loss [24]. A more recent systematic review indicated that implant loss was significantly higher for maxillary IOD supported by less than four implants than for IOD supported by four implants [25]. Boven et al. assessed the masticatory ability of splinted implants in IOD and found that it was significantly better than that of un-splinted solitary implants [26]. IOD using un-splinted solitary implants is still controversial for maxillary edentulous patients due to implant ability to endure force in poor bone quality and quantity as well as the unfavorable biomechanics of the anterior maxillary ridge [12,27]. However, despite these concerns, IOD using un-splinted solitary implants might be considered as a favorable treatment with advantages of easy maintenance, less sensitive technique and lower cost. Even though there are controversies, treatment modality of IOD using un-splinted solitary implants in maxilla has been conducted and reported.

Zou et al. verified that rigid double-crown implant prostheses connected to removable dentures showed good results in fully maxillary edentulous patients and that implant survival rates of double crowns were not significantly different from bar type IOD in the maxilla [28]. However, double-crown prostheses required expensive maintenance and showed high rates of prosthetic complications [29]. Therefore, double-crown implant prostheses with removable dentures are not often used.

To overcome many of the problems described above, we may design a new treatment modality for maxillary edentulous patients: a combination of removable partial dentures with implant crowns. These implant crowns are surveyed for rests, retentive arms, and proximal plates of removable partial dentures (RPDs) as abutments. We define this new treatment modality as implant-crown-retained removable partial dentures (IC-RPD) in this study. By fabricating a few implant crowns and placing them at specific areas (usually canine or premolar positions) that had sufficient alveolar ridge, we could avoid extensive surgical interventions such as sinus lift, bone augmentation, or zygomatic surgery in posterior ridge of maxilla. In such cases, clinicians need only to choose the implant position where residual alveolar bone is suitable for simple implant surgery. Another edentulous area was restored with tissue supported RPD to avoid long fixed prostheses cantilever arm, which might cause unfavorable force. Additionally, the prosthodontic procedures necessary to apply surveyed crowns and RPD are well known by clinicians, therefore, treatment procedures and maintenance are relatively easy for IC-RPD.

For decades, extensive studies on implants combined with removable dentures were performed but were usually focused on IOD using ball, locator, or magnet attachments under the denture base for partially edentulous patients or fully mandibular edentulous patients. There were no long term studies of IC-RPDs for maxillary edentulous patients. By using the new concept of placing implants in strategic positions and restoring them as splinted surveyed crowns with retentive arms, implants could be made more rigid to resist loading and dislodgement in edentulous ridges by reducing the length of the free end saddle [30]. Therefore, we investigated IC-RPDs closely as a potential new treatment modality.

In this study, we evaluated IC-RPDs and compared them to IODs in maxillary edentulous patients. Variables that influence survival and marginal bone loss (MBL) of implants such as first year pathologic condition, location of implants (anterior vs. posterior), age (older than 65 years vs. younger than 65 years), and sex were analyzed in both treatment modalities. The functional/esthetic satisfaction of patients and prosthetic complications after treatments were also observed.

## 2. Materials and Methods

### 2.1. Study Protocol and Eligibility Criteria

Our study sample included 47 maxillary edentulous patients who were treated with IC-RPD or IOD between January 2012 and July 2020 at Seoul National University Dental Hospital and S Leader Dental Clinic in South Korea. This study was authorized by the Institutional Review Board of Seoul National University Graduate school of Dentistry (No. S-D20200040). All patients included in this study were treated by surgical or prosthodontic specialists and visited for periodic recall checks.

Of the 47 patients, we ultimately included 38 (22 men, 16 women) patients and 145 implants in the study. All patients included in this study were treated by surgical or prosthodontic specialists and visited the clinic for periodic recall checks. Patients with systemic diseases (e.g., diabetes or osteoporosis) affecting implant prognosis and patients with any conditions that contraindicated denture recall were excluded. The study sample was divided into two groups: edentulous patients with IOD (i.e., implant overlay complete dentures) and edentulous patients with IC-RPD with splinted implant surveyed crowns.

Following our clinical chart, inclusion criteria for the placements of 145 implants were: (1) adequate bone to accommodate two to four implants over the arch; (2) no severe systemic problems, fair health, and the ability to undergo a surgical procedure under local anesthesia; (3) no drug or alcohol abuse (a smoking cessation program was provided to smokers before treatment); and (4) no unrealistic demands regarding treatment outcome. To estimate implant survival, we evaluated implants according to the Pisa consensus statement from the International Congress of Oral Implantologists (ICOI) Conference 2007 [31]. An implant was considered to have survived if the implant and its superstructure remained in place and functioned normally at the final observation. If any symptoms (e.g., pain on function, mobility, severe radiographic bone loss, uncontrolled exudate, or extraction) were present, the implant was classified as a failure.

### 2.2. Data Collection and Analysis

All implants in IC-RPDs were placed in anterior (canine or premolar) positions to avoid extensive surgery, e.g., lateral sinus floor elevation or bone augmentation, except for one implant that was placed in the left first molar because of consistent previous surgery failures. According to the Kennedy-Applegate classification, only Class I IC-RPD (also Class I with modification 1) was included in our study (Table 1). Major connectors of IC-RPDs were broad palatal coverage or full palatal coverage according to the number and position of implant fixed prostheses. Canine or mesial rest seats were positioned in terminal abutments and all direct retainers were circumferential casted clasps with compensational arms.

**Figure 1:**
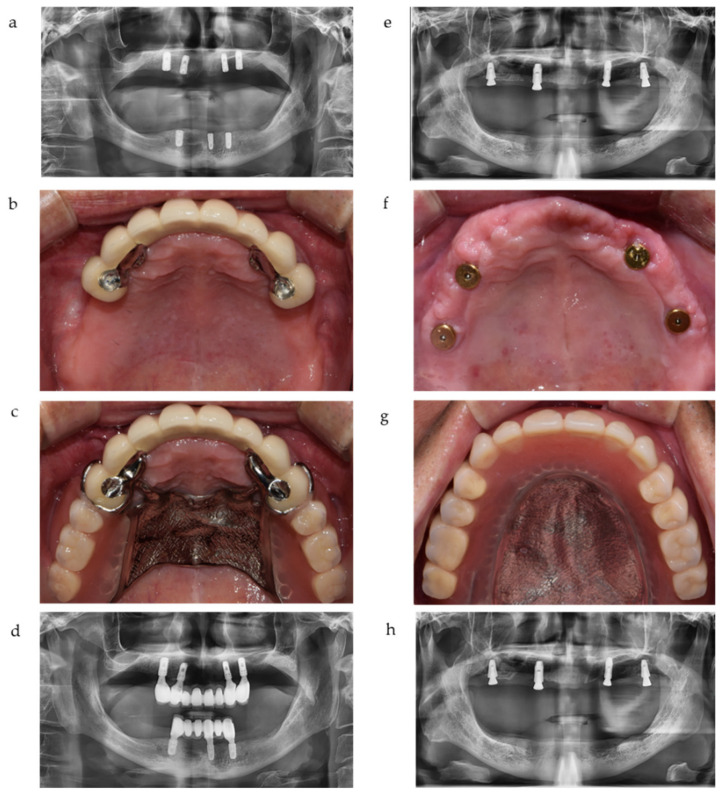
Representative cases of implant-crown-retained removable partial dentures (IC-RPD) and implant overdentures (IOD) in this study. (**a**) Implants were placed in anterior positions due to anatomical limitations. (**b**) Intraoral view of IC-RPD case. (**c**) Delivery of IC-RPD. (**d**) Panoramic radiograph of IC-RPD case. (**e**) Four implants were placed with lateral sinus floor elevation and bone augmentation. (**f**) Intraoral view of IOD case. (**g**) Delivery of IOD. (**h**) Panoramic radiograph of IOD case.

In most cases of IOD, two anterior implants (canine position) and two posterior implants (first molar position) were used for better force distribution. These implants were attached by magnets beneath the IOD as shown in Figure 1. Clinical and radiographic assessments were performed on a total of 145 implants during the observation period.

All 145 implants in this study were bone level internal type (Table 2): 120 were 4–4.5 mm in diameter (with 10 mm or 11.5 mm of length) and 25 were 4.8–6 mm in diameter (with 8.5 mm or 10 mm of length). All 20 IC-RPDs were installed with 74 implant-supported porcelain fused metal (PFM) surveyed crowns and 18 IOD with 71 solitary implants attached to magnets. The follow-up period in this study ranged from 12–147 months (mean 47.1 months).

At the time of prosthesis delivery, an intraoral evaluation of occlusion was performed. To restore edentulous maxilla with Kennedy Class Ⅰ IC-RPDs and IODs, centric relationship was determined by bimanual manipulation and centric occlusion was evaluated by using both 8 μm metal foil (Hanel shimstock, Hager Worldwide, Idaho, USA) and double-sided occlusal masking film (Accufilm, Parkell inc., Brentwood, NY, USA). Maintenance instructions as well as oral and written presentations of each patient’s recall schedule were prepared. Follow-up was conducted annually for all patients for 1–13 years. The following evaluations were made during follow-up: (1) implant survival; (2) radiographs of implant MBL; (3) patient reported oral measurements (PROMs) at 6-month recall check; and (4) prosthetic complications.

The main outcome of this study was cumulative implant survival rate (CSR). The implant survival criteria we used followed the Pisa consensus statement of the ICOI Conference in 2007 [31]. Implants were considered to have survived if the implant and its superstructure were functioning normally at the final observation.

**Figure 2:**
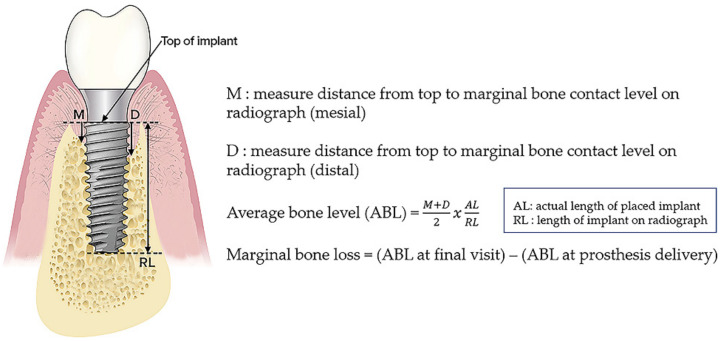
Definition of MBL (marginal bone loss) around implants.

Peri-implant bone resorption was evaluated with annual intraoral radiographs, using digitized panoramic and periapical radiographs. To eliminate bias, all radiographic data was collected and categorized by chart number order regardless of treatment modalities and evaluations were randomly conducted by a single examiner (SYY) according to the same criteria twice. The Intraclass Correlation Coefficient (ICC) value is the reliability calculated by the raters’ measurements. The ICC means reproducibility if the test is repeated several times. Therefore, for reliability of measurement in this study, ICC values were statistically analyzed. Radiographs taken during the final recall visit were used to determine the peri-implant bone level as the distance between the platform of the implant and the level of the adjacent osseous crest on the mesial and distal aspects, respectively. Based on the actual length of the implants, the actual bone level was calculated by a proportional equation [32]. We defined MBL as the mean value of bone resorption in the mesial and distal aspects (Figure 2).

In this study, we observed MBL around implants based on multiple variables such as first year pathologic condition, location of implant placed, age, and sex. The current guidelines for the diagnosis of peri-implantitis were defined by the 2017 World Workshop on the Classification of Periodontal and Peri-implant Diseases and Conditions [33] as follows: (1) presence of bleeding on probing (BOP) and/or suppuration; (2) increased probing depth (PD); and (3) presence of detectable bone loss exceeding measurement error (mean 0.5 mm) with radiographically observed first year pathologic condition. However, opinions vary regarding the best means to define peri-implantitis. Ramanauskaite et al. suggested a rationale for the diagnosis of peri-implantitis [34], and many authors follow the consensus from the First European Workshop suggesting criteria defining implant success as MBL of less than 1.5 mm during the first year after the insertion of the prosthesis and thereafter less than 0.2 mm annual bone loss [35]. Other sources define changes ≥2 mm at any time point during or after the first year as pathologic (i.e., peri-implantitis) [36,37,38]. Overall, in this study, when the MBL was greater than 1.5 mm with increased PD, BOP, and/or suppuration, we diagnosed peri-implantitis [39].

Patient quality of life and satisfaction are the main considerations when choosing treatment modalities [40]. In this study, we examined PROMs after IC-RPD and IOD treatment according to visual analog scales (VAS) of 1–5, in which 1 was the least favorable. Using questionnaires, we asked patients to rate (1) esthetic satisfaction before and after the prosthesis procedure and (2) functional satisfaction before and after the prosthesis procedure. Satisfaction levels were recorded after IC-RPD or IOD prosthesis insertion (usually at the 6-month follow-up).

Finally, we collected all data regarding occlusion and technical complications after prosthesis delivery from clinical charts. All chart records were reviewed to identify complications associated with RPD or implant surveyed crowns in IC-RPDs, and complete denture (CD) or implant attachments in IODs. Prosthetic complications were classified into five categories: (1) denture: fractures or deformations of denture components followed by repair or fabrication of new dentures; (2) implant: screw loosening or fractures; (3) implant surveyed crowns in IC-RPD: dislodgement of prostheses or veneer porcelain fracture in PFM; (4) magnet attachments in IOD: mobility, dislodgement or loss; and (5) tissue: sore spots or crestal bone resorption due to denture base.

### 2.3. Statistical Analysis

All data were evaluated using SPSS version 23 (SPSS Inc., Chicago, IL, USA). In order to analyze the implant CSR, the Kaplan & Meier method was used with a log rank (Mantel-Cox) test to compare variables. The time interval criterion for implant failure and implant MBL was defined as the time difference between delivery date of the prosthesis and complication occurrence date and/or observation end date. We used the Kruskal-Wallis test to identify differences in implant MBL according to treatment modality (IC-RPD vs. IOD), first year pathologic condition, location of implant placed, age, and sex, and then conducted the Mann–Whitney test using those results. In order to define changes in implant MBL through time (before loading, at year 1, at year 2, and at final recall check) according to first year pathologic condition, we applied a linear mixed model, and for posterior comparisons we used the t-test with significance levels adjusted by Bonferroni’s method (*p* = 0.0125). To confirm reliability of measurement on implant MBL, ICC was analyzed at 95% confidence interval in this study.

In addition, we surveyed patients to detect significant functional or esthetic improvements after treatment using the Wilcoxon signed rank test, and also initially applied the Kruskal–Wallis test to determine differences in PROM variables. We made final comparisons of the results derived using the Mann–Whitney test.

## 3. Results

### 3.1. Implant Survival Analysis

In comparisons of survival rates according to treatment modalities, two implant surveyed crowns from IC-RPD and 21 implants from IOD failed, resulting in survival rates of 97.3% for IC-RPD and 70.4% for IOD. There was a significant difference in survival rate by treatment modality (*p* < 0.001) as shown in Table 3. During the observation period, 23 implants of 145 failed for a total survival rate of 84.1%. Table 4 includes specific information for the 23 failed implants.

**Figure 3:**
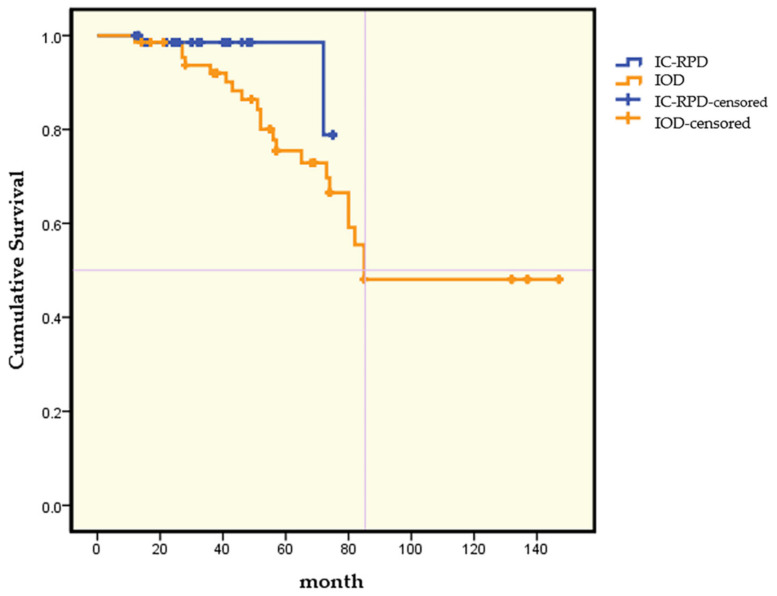
Kaplan–Meier survival curves according to treatment modality (IC-RPD vs. IOD).

Kaplan–Meier survival curves according to treatment modalities are illustrated in Figure 3. At year 1, both showed 98.6% CSR of implants but as illustrated in Figure 3, the difference widened with time. At year 3, for ISC-PRD, the CSR of implants remained at 98.6% while the CSR of implants in IOD decreased to 92%. At month 74, the CSR in IC-RPD was 78.9% and for IOD it was 66.5%. For IOD, the CSR of implants decreased to 55.4% at month 82.

As shown in Table 4, two failed implants from IC-RPD were placed at the canine or premolar positions and occluded with the remnant natural tooth connected to mandibular RPD. One previously supported full maxillary fixed prostheses but was no longer available to support fixed prostheses after other posterior and anterior implants were removed due to severe bruxism. Therefore, this implant was converted to an implant surveyed crown for IC-RPDs with cingulum rest/Akers’ clasps, and was removed after 72 months of function. The other implant failed after 14 months and there was no peri-implantitis sign according to the clinical chart. Based on the radiolucency around the implant threads when it was removed, we assumed that excessive loading due to bruxism was the main reason for failure. Both patients who had failed implants in IC-RPD showed uncontrolled severe attrition in dentures.

Twelve of 21 failed implants in IOD were from only three patients whose implants were all removed. Four patients previously had full maxillary fixed prostheses but after constituent failures ended up with IOD with four solitary attached implants beneath to the denture base. After a mean 26 months of use of IOD, MBL increased severely with suppuration as shown in Table 4 and we therefore extracted them. Another eight failed implants in IOD were associated with horse-shoe type major connector maxillary dentures. Of four IOD with horse-shoe palatal coverage, only one IOD remained functional after 137 months. However, no posterior placed implants in IOD with sinus bone augmentation failed.

In the present study, all opposing dentitions were natural teeth, fixed implant prostheses, or IOD. There were no significant differences in implant failure according to opposing dentition type regardless of treatment modalities (*p* = 0.866; 0.842 for IC-RPD group and 0.131 for IOD group respectively).

There was no association between implant failure and age (*p* = 0.156), as shown in Table 3. The position of implants (anterior or posterior) did not affect failure rate (*p* = 0.223). However, implant survival was significantly greater in males than females (*p* = 0.027).

### 3.2. Implant Marginal Bone Loss Analysis

Table 5 shows the analysis of MBL surrounding implants. The implant MBL of all patients was 1.08 ± 1.02 mm at year 1 and 2.18 ± 1.83 mm at final check-up (13 to 147 months). ICC of MBL measurement was 0.97 at delivery, 0.982 at year 1, and 0.987 at final check-up. All MBL measurements exhibited an excellent reliability, based on 95% confident interval of the ICC estimation.

**Figure 4:**
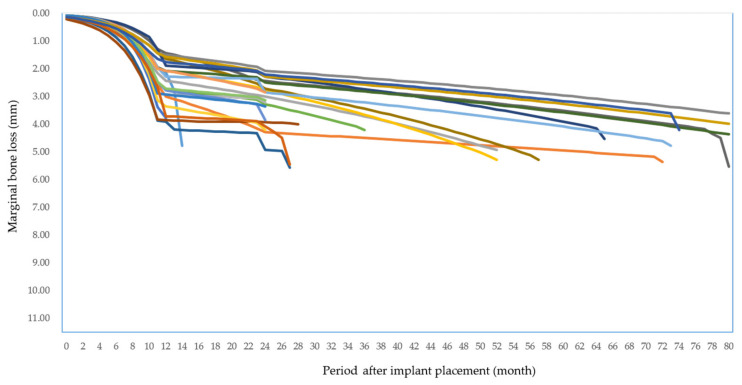
Marginal bone loss of failed implants.

The implant MBL of most failed implants showed early bone loss as illustrated in Figure 4.

**Figure 5:**
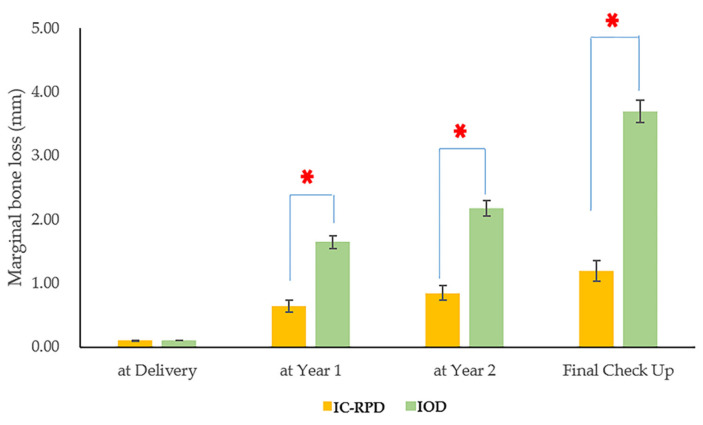
Marginal bone loss change through time according to treatment modality. The red asterisks indicate significant differences between IC-RPD and IOD groups (*p* < 0.0125; adjusted by Bonferroni’s method). Average time of final check-up (i.e., observation end date) was different for IC-RPD (32.3 months) and IOD (63.4 months) groups. Asterisks indicated *p* < 0.001.

The implant MBL was significantly different based on treatment modality over time (*p* < 0.001). The implant MBL of IOD was significantly higher at final recall check (*p* < 0.001) as shown in Figure 5.

MBL of implants showed significant differences based on treatment modality and first year pathologic condition (i.e., peri-implantitis) (*p* < 0.001). Higher MBL levels were seen in patients over 65 years of age (*p* = 0.029) while position of placed implants (*p* = 0.397) and sex (*p* = 0.343) showed no significant differences as depicted in Table 6.

### 3.3. PROMs

**Figure 6:**
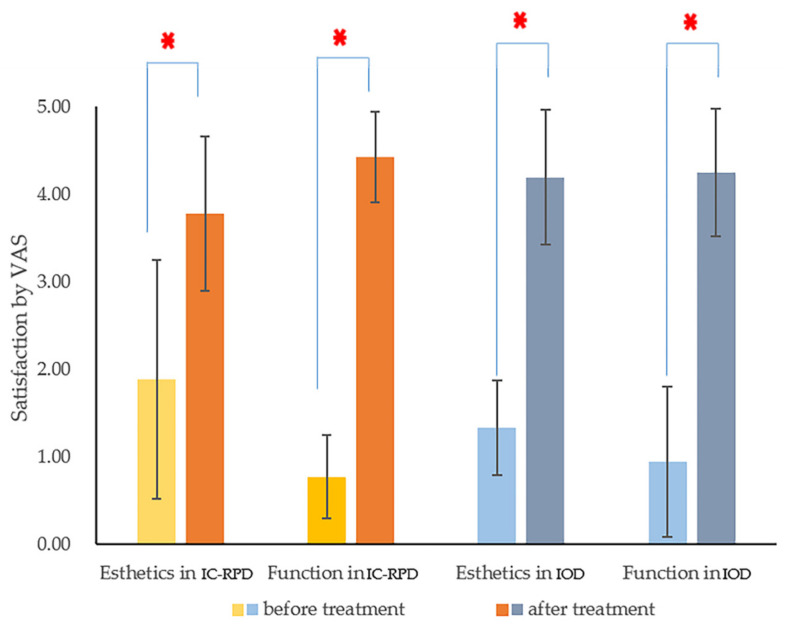
Comparison of satisfaction (esthetic and function) rates by visual analog scales (VAS) before vs. after prosthetic treatment. The Wilcoxon-signed ranks test showed there were significant differences before and after treatment in both esthetics and function regardless of treatment modality (*p* < 0.05). Asterisks indicated *p* < 0.001.

**Figure 7:**
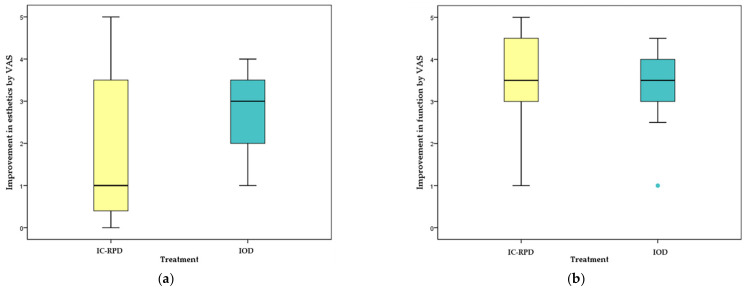
Esthetic and functional improvement by VAS according to treatment modality (IC-RPD vs. IOD). (**a**) Esthetic satisfaction showed significantly different results according to treatment modality; the IOD group showed statistically higher measured values (*p* = 0.041). (**b**) Functional improvement showed no differences (*p* > 0.05) between groups. Green dot indicated only one patient with VAS value of 1 after delivery of IOD.

For both IC-RPD and IOD groups, patient satisfaction improved significantly (*p* < 0.001) after the delivery of new prostheses according to the Wilcoxon signed rank test (Figure 6). However, patients who wore IC-RPD in the maxilla were not as satisfied with the esthetic improvement as patients with IOD. Therefore, the VAS of esthetic appearance improvement was significantly higher in the IOD group (*p* = 0.041 <0.05). Mastication ability showed significant improvement after prosthesis installment, however, there was no significant difference between treatment modalities (*p* = 0.346) (Figure 7).

### 3.4. Prosthetic Complications

The complications in both treatment modalities were divided into four categories each (Table 7 and Table 8). After the delivery of the prosthesis, the most common mechanical complication in IC-RPD was the adjustment of clasps.

Three of 20 patients experienced dislodgement of surveyed implant crowns due to washout of temporary cement, but these problems were easily resolved by re-cementation. All cases where dislodgement of surveyed crowns occurred were long-span six-unit bridges with retentive clasps. However, the mechanical complications of IOD showed much higher incidences. In IOD, wear or dislodgement of magnet attachments was the most frequent complication, with a total of four patients losing attachments. In addition, 27% of patients experienced denture base fracture or implant screw loosening. All complications were resolved by repairing or changing the components.

## 4. Discussion

We investigated the survival rates and MBL of implants according to removable prostheses treatment modalities in maxillary edentulous patients. Restorations with IC-RPD or IOD in this study were determined by cost and patients’ opinions. Due to the relatively high cost of fixed prosthesis in IC-RPD compared to attachments in IOD, even though some patients wanted to restore few implants with crowns to comfort dejection, they could not choose IC-RPD but still wanted IOD for better support and retention instead of CD. Similarly, patients who wanted IC-RPD could not choose full arch fixed prostheses due to relatively high cost of fixed prostheses and need for extensive surgeries.

The overall implant survival rate of combined IC-RPD and IOD groups in this study was 84.1% (up to 147 months), which is consistent with previously reported implant survival rates of 86.3% by Fabrro et al. and 85% by Rammelsberg et al. [41]. There were significant differences in implant survival rates according to treatment modality: IC-RPD (97.3% during a mean 32.3 months) compared to IOD (70.4% during a mean 64.7 months), even though observation time differed according to treatment modality (*p* < 0.001). The Kaplan–Meier analysis showed that in 3 years the CSR of implants was 98.6% in the IC-PRD group and 92% in the IOD group. This agrees with the results of the 2015 Clinical Oral Implants Research consensus that in the edentulous maxilla, removable prostheses with four implants were associated with high implant survival estimates at 5 years [42]. However, after 6 years, implant CSRs in the present study decreased to much lower values, which were 78.9% for the IC-RPD group and 66.5% for the IOD group.

Maxillary trabecular bone is not naturally conducive to primary implant stability, and bone resorption patterns can create unfavorable occlusal forces. Previous studies reported that lower maxillary implant survival rates were correlated with decreased quality and quantity of bone, implant angulation that followed the resorption pattern of bone, and increased abutment length caused by thickened maxillary mucosa [43,44]. To increase survival rates by balancing forces, we made splinted maxillary implant crowns in the present study. Grossmann et al. recommended that implants should be splinted to improve stress distribution when they are off-axis, including during canine restoration and when remnant natural tooth stops are reduced [45]. Therefore, our findings of significantly higher survival rates of splinted implant crowns in the IC-RPD group compared to solitary implants in the IOD group led us to hypothesize that splinting with approximate implant crowns in IC-RPD allowed implant forces to be distributed more appropriately and therefore reduced failures.

Table 4 includes specific information for failed implants in IOD. Alsrouji et al. reported severe bone resorption of premaxillary residual ridges in IOD due to the high strain and total deformation of bone under off-set force caused by the unfavorable axes of implants [46]. Failed implants beneath the IOD of the present study showed similar characteristics, with implants placed in the premaxilla failing earlier than implants in the posterior region, even the though numbers of failed anterior and posterior implants were similar.

A previous study showed when the opposing dentitions were RPD or CD, there were no implant failures, resulting in a 100% survival rate [47]. In the present study, all opposing dentitions were natural teeth, fixed implant prostheses, or IOD, and overall implant survival rate was 84.1%. The differences might be due to the occlusal forces.

Adell reported that the MBL of successful implants disappeared after 1 year of abutment connection, so prognosis should be assessed after 1 year, and MBL at that time was 1.2 mm [48]. There was another suggestion that MBL of functional implants should not exceed 2 mm [36,37,38]. Alberktsson et al. defined MBL of less than 1.5 mm during the first year as successful [35]. Recommended guidelines for determining the normal range of MBL remain controversial. In this study, we defined MBL greater than 1.5 mm as pathological and affected by peri-implantitis.

At the first year, 11% implants in the IC-RPD group and 50% of implants in the IOD group showed pathologic peri-implantitis (MBL greater than 1.5 mm with increased PD, BOP, and/or suppuration), 29.7% of 145 implants overall. MBL of implants with peri-implantitis at year 1 in IC-RPD and IOD groups showed significantly greater bone loss over time according to the post hoc T test after linear model analysis (significance level < 0.0125, or the *p* value of Bonferroni’s correction). We conclude that when MBL of implants with peri-implantitis at year 1 showed significantly greater bone loss they were associated with higher failure rates (*p* < 0.001).

In the present study, the MBL in 145 implants was 1.58 ± 1.02 mm, similar to previous studies [49,50]. When we compared MBL according to treatment modalities (IOD vs. IC-RPD), there was a significant difference between groups. A previous study by Bae et al. reported that the MBL around implants was significantly higher in solitary implants attached beneath IOD (1.99 ± 0.7 mm) than in splinted implants used as surveyed crowns in IC-RPD (1.44 ± 0.57 mm) [51]. Our result was in accordance with these previous studies. The overall MBL in the IC-RPD group was significantly lower than in the IOD group (*p* < 0.001). We hypothesized that the implants are connected to and fixed by splinted crowns, which reduces stress, causing micro-damage by efficiently dispersing the load generated during mastication [52]. However, the mean observation period in the IOD group (63.4 months) was much longer than in the IC-RPD group (32.3 months), and therefore further cross-sectional studies with longer observation times are necessary.

In the present study, many cases with peri-implantitis at year 1 had common features such as immediate implant placements in narrow anterior regions or occluding dentition being natural teeth or implants. When the level of immediate implant placement was not deep enough to facilitate the bone remodeling healing process, there could be loss of bone around implants during healing of the narrowing buccal plate [53]. If implants occluded with opposing natural teeth or implants as the only stop, excessive loading that could lead to micro fractures around implants could result in severe bone loss or failure. Therefore, for successful rehabilitation of patients treated by IC-RPD or IOD modalities, occlusal adjustment should be performed carefully while focusing on appropriate stress distribution by strategic implant positioning as well as designs intended to prevent excessive loading.

We investigated PROMs (assessing satisfaction with functional and esthetic changes after treatment) using a VAS scale at the first recall check (in most cases at 6 months). Patients were satisfied with their new implant prostheses regardless of the treatment modality and stated that they could eat better and looked younger. There was no difference between groups (IC-RPD or IOD) in functional improvement (*p* = 0.346), but there was a significant difference in perceived esthetic improvement (*p* = 0.041). Some patients were more satisfied with dentures, but the greatest increases in measurement values (differences of 5 points from 0 before treatment to 5 after treatment) for esthetics were all in the IC-RPD group among patients treated by anterior long span bridge (Br). For them, even without RPD, appearance after prosthesis placement was deemed good due to restored lip support.

Patients who received IC-RPD treatment with few implants expected their anterior teeth to be restored. However, cross arch anterior fixed prostheses could not be applied to all of these patients due to the lack of alveolar bone at the anterior position (premaxilla). When there were no implants placed at the four anterior teeth, long-span cross-arch Br with canine or premolar implant abutments were difficult to install, due to the anterior and posterior spread (AP spread) and cantilever. English et al. proposed a very reasonable rule of thumb for determining the length of cantilever, that it should be to 6–8 mm (0.7-8 fold of AP spread) due to low bone density [54]. Khorshid et at. found that a cantilever extension to AP spread with 1:3 ratio induced the least crestal bone height loss around each implant in the maxilla [55]. Therefore, in cases with unfavorable pre-maxillary bone, we placed implants symmetrically at only canine or premolar positions, and splinted them without anterior pontics.

Fabrication of IC-RPD might be harder than IOD. In this study, abutments for all implant surveyed crowns were manufactured in a customized manner due to the unfavorable axis followed by severe bone loss. Complicated surveying for implant crowns was also needed with poor interdental relationship because patients who had severe resorption of maxilla were indicated for IC-RPDs in this study. Resultantly, the surveyed crowns had unfavorable crown/implant ratio as well as bulged height of contour.

However, the overall frequency of mechanical complications was higher in the IOD group. In the IC-RPD group, mechanical complications included three cases of dislodgement of surveyed crowns and 19 cases of loosening of clasp arms. The dislodgement of crowns occurred because we cemented them with temporary implant cement (Premier Implant Cement, Plymouth Meeting, PA, USA.) and dislodgement occurred only when they occluded with natural teeth or implants. To address loosening of retentive arms due to repetitive insertion of dentures, we adjusted clasp arms with a utility appliance. Such complications could be resolved easily.

In IOD, most mechanical complications occurred in attachments. All attachments used in the IOD group were flat type magnets (MGT5515, Dentium Co., Seoul, Korea) because they alleviate detrimental lateral stresses to the fixture due to very low attractive horizontal forces [56]. The influence of implant inclination on unfavorable lateral forces is limited in magnet attachment systems [57]. Use of magnet attachments is easy due to reduced vertical dimension. Despite such advantages, in the present study, 27 magnets (44% patients in the IOD group) were re-attached beneath eight dentures and 16% of patients in the IOD group lost their attachments. In addition, 27% patients in the IOD group experienced denture base fractures that needed to be repaired in the lab. Most fractures occurred around implant attachments and we therefore hypothesized that lack of clearance due to the height of the attachment or stronger retentive and dislodging forces around the implant fulcrum affected the fracture. Other mechanical complications, e.g., screw loosening, were often observed to complicate the maintenance of IOD (Table 8). The costs associated with maintenance of attachments in IOD are high [58]. Among biological complications, several studies reported that implants in the IOD group also showed higher frequency of calculus deposition and clinical complications compared to implants in IC-RPD [51,59]. IC-RPD is easier to maintain than IOD.

The IC-RPD group had the advantage of lower decrease in retention compared to the IOD group. The retentive forces of the clasp arm can be adjusted relatively easily by the dentist, and if there is no defect in the laboratory process, no adjustments are needed for relatively long periods. However, as we found, the frequency of requirement maintenance of the implant attachment was high in the IOD group due to wear that occurs during the attachment and detachment of the denture, as well as functional load during mastication [60].

Regarding the design of IOD, a recent systematic review concluded that a palate-less design supported by 4 to 6 implants was successful for the treatment of maxillary edentulism [61]. However, palate-less (horse-shoe) CD designs might lead to insufficient support and retention due to the absence of the posterior palatal seal. In this study, 17 of the 18 IODs had full palatal coverage and only 1 IOD had horseshoe design at final recall check because 3 of 4 horse-shoe IODs were refabricated to full palatal coverage to improve the stress distribution to the tissue when patients started to lose implants. However, because severe MBL around implants had already occurred before the new full palatal coverage IOD was fabricated, 3 refabricated full palatal coverage IODs ended up with CD.

There are various suggestions regarding locations of implant placement for implant assisted removable prostheses. A previous study showed that implant placement in the residual alveolar ridge at the second molar decreases stresses around teeth [62]. Yang et al. evaluated posterior implant supported fixed dental prostheses with Class IV RPD in fully maxillary edentulous patients and deemed them a good treatment option [63]. However, posterior implant position in removable prostheses can lead to excessive forces on implants even if they decrease tissue bound movement [64,65,66,67], and in the maxilla they require additional procedures such as lateral sinus floor elevation.

Del’Arco Pignatta Cunha et al. reported that when the location of implants moved anteriorly from the molar to the premolar area, the force distribution around the abutment and tissue was more favorable [67]. In the theoretical experimental model, as the implant moves to a more posterior position in the distal extension area, the stress on the implants and abutment teeth was increased [68]. In the same context, previous studies found that implants located in the area of the second bicuspid were the least likely to displace the abutment tooth compared with other locations [65,66,67,69]. Another study reported the first premolar was mainly considered to improve stress distribution and retention [70,71,72], while other case reports demonstrated the benefits of placing implants at the anterior region, which fulfilled the functional and esthetic requirements of the patient without jeopardizing the natural teeth [73]. In the maxilla, posterior implant placement often entails maxillary sinus augmentation, which increases costs and surgical time. Implant placement in the premolar region without bone augmentation could be cost effective for surveyed crowns of IC-RPD.

Within the limits of this study, we demonstrated that anteriorly positioned (canine or premolar area) splinted implants used as surveyed crowns for IC-RPD yielded favorable results in severely resorptive maxilla. There are also advantages associated with transformation to fixed implant-supported restorations by adding more implants, in case patients change their minds and decide to restore edentulous ridges with fixed prostheses [64]. And to salvage failed full arch fixed implant prostheses, IC-RPD can be a good option. With the help of implants, edentulous patients may benefit from more supportive, stable, and retentive prostheses. Therefore, clinicians should consider new treatment modalities (IC-RPD) with periodic recall checks and thorough oral hygiene (OH) instruction.

## 5. Conclusions

The survival rates of splinted implant crowns in IC-RPDs were 97.3% while solitary implants attached beneath IODs was 70.4% (*p* < 0.001). There was a significant higher MBL of implants for the IOD group (3.31 ± 1.71 mm) than IC-RPD group (1.12 ± 1.19 mm) (*p* < 0.001). PROMs showed statistically significant higher improvement on masticatory function and esthetic appearance in both groups. However, the IOD group showed more frequent prosthetic complications compared to the IC-RPD group. Within the limitations of this study, we conclude that IC-RPD is a viable treatment for maxillary fully edentulous patients with anatomical limitations to avoid extensive surgeries at a reasonable price.

## Figures and Tables

**Table 1:** Kennedy-Applegate classification in the implant-crown-retained removable partial dentures (IC-RPD) group.

IC-RPD	Modification	Implant Position	IC-RPD Number
Class Ⅰ	no modification	anterior	8
	with modification 1	anterior or premolar	12

**Table 2:** Numbers of implants for IC-RPD and IOD and related information.

Treatment Modality	Implant Connection Type	Implant Manufacturer	Implant Diameter	Total
IC-RPD (*n* = 20)	Internal type	Osstem	Regular (4, 4.5 mm)	40
			Regular (5, 6 mm)	6
		Dentium	Regular (4.3 mm)	24
			Regular (4.8 mm)	4
IOD (*n* = 18)	Internal type	Osstem	Regular (4, 4.5 mm)	19
			Regular (5, 6 mm)	8
		Dentium	Regular (4.3 mm)	37
			Regular (4.8 mm)	7

**Table 3:** Survival rates of implants and *p*-values according to variables.

Condition		No. of Implants	Failed Implants	Survival Rate (%)	*p*-Value
Treatment modality	IC-RPD	74	2	97.3	<0.001
	Overdenture	71	21	70.4	
First year pathologic condition	With peri-implantitis	43	22	48.8	<0.001
	Without peri-implantitis	102	1	99.1	
Location of implant	Anterior	72	10	86.1	0.223
	Posterior	73	13	82.2	
Age	under 65	58	5	87.5	0.104
	above 65	87	18	82.9	
Sex	Male	84	9	89.3	0.027
	Female	61	14	77.1	

**Table 4:** Detailed information for 23 failed implants.

Condition	Patients with Failed Implants										
	A	B	C	D	E	F	G	H	I	J	K
Patients Age/Sex	59/F	66/M	72/F	62/M	69/M	77/F	79/F	76/F	78/M	67/F	69/F
**Treatment Modality**	**Implant Surveyed Crown**		**Implant Overdenture**								
Location of implant	#24	#13	#16, 26	#13,16,23,26	#13,16,23,26	#17	#13,16,23,26	#23,27	#14	#13,23	#13
Diameter/length of implant (mm)	4.0/10.0	4.0/11.5	4.0/10	Ant. (4.3/10)	Ant. (4.3/10)	5.0/10	Ant. (4.0/10)	4.0/10	4.0/11.5	4.0/10	4.3/10
				Post. (4.8/10)	Post. (4.8/10)		Post. (5/10)				
Survival period (months)	14	72	52/52	12/36/27/28	80/57/65/62	73	43/80/41/56	46/51	27	85/85	74
Opposing dentition	Ant. Natural tooth + RPD	Ant. Natural tooth + RPD	Overdenture	Overdenture	Natural tooth + Implant	Overdenture	Natural tooth + Implant	IC-RPD	Natural tooth	Natural tooth	Ant. Natural tooth + RPD
Major connector	Full-palatal coverage	Full-palatal coverage	Full-palatal coverage	Full-palatal coverage	Horse-shoe major connector	Full-palatal coverage	Horse-shoe major connector	Horse-shoe major connector	Full-palatal coverage	Full-palatal coverage	Full-palatal coverage
Reason for failure	Pain, mobility	Bone loss, exudate	Pain, exudate	Pain, mobility, exudate	Bone loss, exudate	Bone loss, exudate	Bone loss, exudate	Bone loss, exudate	Pain, mobility	Bone loss, exudate	Bone loss, exudate

Abbreviations: M, Male; F, Female.

**Table 5:** Marginal bone loss (MBL) of implants in IC-RPD and IOD at year 1 and at end date of observation.

	IC-RPD (*n* = 74) (mm)	IOD (*n* = 71) (mm)	Total (*n* = 145) (mm)	*p*-Value
At year 1	0.65 ± 0.95	1.58 ± 0.58	1.08 ± 1.02	<0.0125
At end date of observation	1.12 ± 1.19	3.31 ± 1.71	2.18 ± 1.83	<0.0125

**Table 6:** MBL of implants in IC-RPD and IOD according to variables.

Condition		No. of Implants	Bone Loss (mm)	*p*-Value
Treatment modality	IC-RPD	74	1.12 ± 1.19	<0.001
	Overdenture	71	3.31 ± 1.71	
First year pathologic condition	With peri-implantitis	43	4.20 ± 1.34	<0.001
	Without peri-implantitis	102	1.32 ± 1.33	
Location of implant placed	Anterior	72	2.01 ± 1.73	0.397
	Posterior	73	2.35 ± 1.92	
Age	under 65	58	1.96 ± 1.81	0.029
	above 65	87	2.51 ± 1.81	
Sex	Male	84	2.04 ± 1.76	0.343
	Female	61	2.37 ± 1.92	

**Table 7:** Complications in IC-RPDs with implant crowns.

	Prosthetic Complication	Incidences/Patients	Remarks
Denture	Fracture of RPD clasp	-	-
	Fracture of RPD rest	-	-
	Fracture of artificial teeth		-
	Clasp loosening	19/9	Re-adjustment
Implant	Implant screw loosening	-	-
	implant screw fracture	-	-
Crown	Dislodgement	3/3	Re-cementation (temporary cement loss)
	Crown veneer fracture	-	Repair
Tissue	Sore spot around Major connector	1/1-	Relief
	Denture base sore spot	-	-
	Crestral bone resorption	-	-

**Table 8:** Complications in IOD with magnet attachments.

	Prosthetic Complication	Incidences/Patients	Remarks
Denture	Fracture of artificial teeth	2/1	Repair
	Denture base fracture	8/5	Around implant attachment -> Repair
Implant	Keeper screw loosening	7/5	Retightening with 30N torque
	implant screw fracture	1/1	Change to new screw
Attachment	Mobility or dislodgement	27/8	Attachment change and/or denture relining
	loss of attachment	4/3-	Change to new attachment
Tissue	Sore spot around Major connector	1/1-	Relief
	Denture base sore spot	-	Relief
	Crestal bone resorption	10/6	Relining

## Data Availability

Data sharing is not applicable.
